# (2,2′-Bipyridine-κ^2^ *N*,*N*′)[*N*-(2-oxido-1-naphthyl­idene)threoninato-κ^3^ *O* ^1^,*N*,*O* ^2^]copper(II)

**DOI:** 10.1107/S1600536808012191

**Published:** 2008-04-30

**Authors:** Zhanglei Qiu, Lianzhi Li, Yan Liu, Tao Xu, Daqi Wang

**Affiliations:** a School of Chemistry and Chemical Engineering, Liaocheng University, Shandong 252059, People’s Republic of China

## Abstract

In the title complex, [Cu(C_15_H_13_NO_4_)(C_10_H_8_N_2_)], the Schiff base ligand is derived from the condensation of 2-hydr­oxy-1-naphthaldehyde and l-threonine. The Cu^II^ atom is five-coordinated by one N atom and two O atoms from the Schiff base ligand and by two N atoms from a 2,2′-bipyridine ligand in a distorted square-pyramidal geometry. In the crystal structure, the combination of inter­molecular O—H⋯O and C—H⋯O hydrogen bonds leads to a two-dimensional network.

## Related literature

For related literature, see: Garnovski *et al.* (1993 ▶); Kalagouda *et al.* (2006 ▶); Wang *et al.* (1999 ▶).
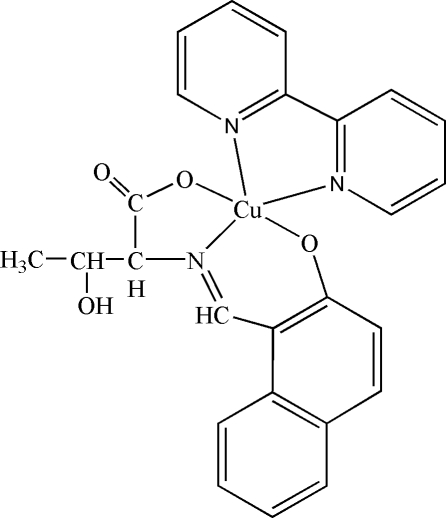


## Experimental

### 

#### Crystal data

[Cu(C_15_H_13_NO_4_)(C_10_H_8_N_2_)]
*M* *_r_* = 490.99
Orthorhombic, 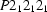

*a* = 9.955 (2) Å
*b* = 12.180 (3) Å
*c* = 18.438 (4) Å
*V* = 2235.6 (9) Å^3^
*Z* = 4
Mo *K*α radiation
μ = 1.01 mm^−1^
*T* = 298 (2) K
0.29 × 0.28 × 0.16 mm

#### Data collection

Bruker SMART APEX CCD area-detector diffractometer
Absorption correction: multi-scan (*SADABS*; Sheldrick, 1996 ▶) *T*_min_ = 0.757, *T*_max_ = 0.855
14242 measured reflections
5278 independent reflections
4243 reflections with *I* > 2σ(*I*)
*R*_int_ = 0.027

#### Refinement

*R*[*F* ^2^ > 2σ(*F* ^2^)] = 0.034
*wR*(*F* ^2^) = 0.088
*S* = 1.04
5278 reflections
300 parameters
492 restraints
H-atom parameters constrained
Δρ_max_ = 0.32 e Å^−3^
Δρ_min_ = −0.54 e Å^−3^
Absolute structure: Flack (1983 ▶), 2229 Friedel pairs
Flack parameter: −0.005 (13)

### 

Data collection: *SMART* (Bruker, 2007 ▶); cell refinement: *SAINT* (Bruker, 2007 ▶); data reduction: *SAINT*; program(s) used to solve structure: *SHELXS97* (Sheldrick, 2008 ▶); program(s) used to refine structure: *SHELXL97* (Sheldrick, 2008 ▶); molecular graphics: *SHELXTL* (Sheldrick, 2008 ▶); software used to prepare material for publication: *SHELXTL*.

## Supplementary Material

Crystal structure: contains datablocks global, I. DOI: 10.1107/S1600536808012191/hy2129sup1.cif

Structure factors: contains datablocks I. DOI: 10.1107/S1600536808012191/hy2129Isup2.hkl

Additional supplementary materials:  crystallographic information; 3D view; checkCIF report

## Figures and Tables

**Table:** 

Cu1—O4	1.925 (2)
Cu1—N1	1.926 (2)
Cu1—N3	2.005 (2)
Cu1—O1	2.0236 (18)
Cu1—N2	2.231 (3)

**Table:** 

O4—Cu1—N1	92.62 (8)
O4—Cu1—N3	91.77 (9)
N1—Cu1—N3	174.65 (9)
O4—Cu1—O1	147.49 (9)
N1—Cu1—O1	82.08 (8)
N3—Cu1—O1	92.57 (9)
O4—Cu1—N2	109.51 (10)
N1—Cu1—N2	104.07 (9)
N3—Cu1—N2	77.29 (10)
O1—Cu1—N2	102.88 (9)

**Table 2:** Hydrogen-bond geometry (Å, °)

*D*—H⋯*A*	*D*—H	H⋯*A*	*D*⋯*A*	*D*—H⋯*A*
O3—H3⋯O2^i^	0.82	1.99	2.808 (3)	178
C5—H11⋯O2^i^	0.93	2.49	3.311 (3)	147
C12—H27⋯O2^i^	0.93	2.50	3.431 (4)	174
C14—H25⋯O3^ii^	0.93	2.52	3.445 (4)	177

Symmetry codes: (i) 
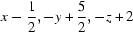
; (ii) 
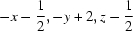
.

**Fig. 1.**
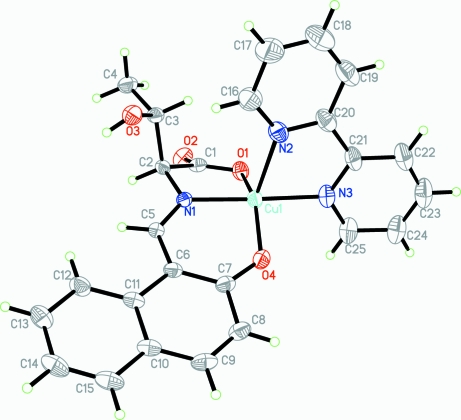
Molecular structure of the title compound. Displacement ellipsoids are drawn at the 30% probability level.

**Fig. 2.**
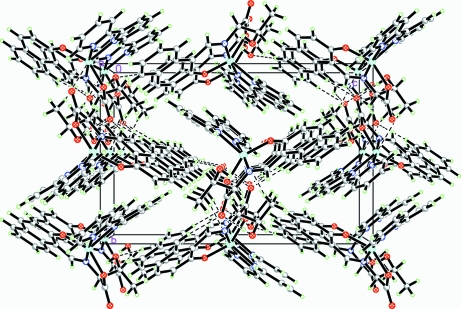
Packing diagram of the title compound with hydrogen bonds shown as dashed lines.

## supplementary crystallographic information

### Comment

Amino acids and their derivatives are very important in molecular biology because of their roles in biochemical reactions. Schiff base complexes have continued to play the role of the most important stereochemical models in main group and transition metal coordination chemistry with their easy preparation and structural variation (Garnovski *et al.*, 1993). So efforts have been made to synthesize and characterize amino Schiff base complexes with transition metals and more and more these new complexes have been reported (Kalagouda *et al.*, 2006; Wang *et al.*, 1999). Herein, we report the synthesis and crystal structure of a copper(II) complex with a tridentate Schiff base ligand derived from the condensation of 2-hydroxy-1-naphthaldehyde and L-threonine.

In the title compound (Fig. 1), the Cu^II^ atom is in a distorted square-pyramidal coordination geometry, defined by one N and two O atoms from the Schiff base ligand and two N atoms from a 2, 2'-bipyridine ligand. The Cu atom deviates from the basal equatorial plane (formed by O1, N1, O4 and N3) by 0.230 (2) Å toward N2 atom, with a significantly longer Cu1—N2 bond distance (Table 1). The Cu1—N2 bond deviates greatly from the right position to close the Cu1—N3 bond [the bond angle of N3—Cu1—N2 is 77.3 (1)°]. The bipyridine ligand deviates from planarity, with an angle of 11.7 (1)° between the two pyridyl rings. The least-square plane of the bipyridine ligand is approximately perpendicular to the basal equatorial plane [dihedral angle 100.3 (2)°]. In the crystal, the combination of intermolecular O—H···O and C—H···O hydrogen bonds (Table 2) leads to a two-dimensional network structure (Fig. 2).

### Experimental

L-Threonine (0.119 g, 1 mmol) was dissolved in hot methanol (10 ml), which was then added to a methanol solution (3 ml) of 2-hydroxy-1-naphthaldehyde (0.172 g, 1 mmol). The mixture was stirred at 323 K for 2 h. Subsequently, an aqueous solution (2 ml) of cupric acetate monohydrate (0.200 g, 1 mmol) was added dropwise and stirred for 2 h. A methanol solution (5 ml) of 2,2'-bipyridine (0.156 g, 1 mmol) was added dropwise and stirred for 4 h. The solution was held at room temperature for 10 d and blue block crystals suitable for X-ray diffraction were obtained.

### Refinement

H atoms were positioned geometrically and refined as riding atoms, with C—H = 0.93 (aromatic), 0.98(CH) and 0.96(CH_3_) Å, and O—H = 0.82 Å, and with *U*_iso_(H) = 1.2*U*_eq_(C) or 1.5*U*_eq_(C,*O*) for methyl and hydroxyl groups.

### Crystal data

**Table:**

[Cu(C_15_H_13_NO_4_)(C_10_H_8_N_2_)]	*F*_000_ = 1012
*M**_r_* = 490.99	*D*_x_ = 1.459 Mg m^−^^3^
Orthorhombic, *P*2_1_2_1_2_1_	Mo *K*α radiation λ = 0.71073 Å
Hall symbol: P 2ac 2ab	Cell parameters from 5604 reflections
*a* = 9.955 (2) Å	θ = 2.3–26.1º
*b* = 12.180 (3) Å	µ = 1.01 mm^−^^1^
*c* = 18.438 (4) Å	*T* = 298 (2) K
*V* = 2235.6 (9) Å^3^	Block, blue
*Z* = 4	0.29 × 0.28 × 0.16 mm

### Data collection

**Table:**

Bruker SMART APEX CCD area-detector diffractometer	5278 independent reflections
Radiation source: fine-focus sealed tube	4243 reflections with *I* > 2σ(*I*)
Monochromator: graphite	*R*_int_ = 0.027
*T* = 298(2) K	θ_max_ = 28.3º
φ and ω scans	θ_min_ = 2.0º
Absorption correction: multi-scan(SADABS; Sheldrick, 1996)	*h* = −13→13
*T*_min_ = 0.757, *T*_max_ = 0.855	*k* = −14→16
14242 measured reflections	*l* = −20→24

### Refinement

**Table:**

Refinement on *F*^2^	Hydrogen site location: inferred from neighbouring sites
Least-squares matrix: full	H-atom parameters constrained
*R*[*F*^2^ > 2σ(*F*^2^)] = 0.034	*w* = 1/[σ^2^(*F*_o_^2^) + (0.0472*P*)^2^] where *P* = (*F*_o_^2^ + 2*F*_c_^2^)/3
*wR*(*F*^2^) = 0.089	(Δ/σ)_max_ = 0.001
*S* = 1.04	Δρ_max_ = 0.32 e Å^−^^3^
5278 reflections	Δρ_min_ = −0.54 e Å^−^^3^
300 parameters	Extinction correction: none
492 restraints	Absolute structure: Flack (1983), 2229 Friedel pairs
Primary atom site location: structure-invariant direct methods	Flack parameter: −0.005 (13)
Secondary atom site location: difference Fourier map	

### Special details

**Table:**

Geometry. All e.s.d.'s (except the e.s.d. in the dihedral angle between two l.s. planes) are estimated using the full covariance matrix. The cell e.s.d.'s are taken into account individually in the estimation of e.s.d.'s in distances, angles and torsion angles; correlations between e.s.d.'s in cell parameters are only used when they are defined by crystal symmetry. An approximate (isotropic) treatment of cell e.s.d.'s is used for estimating e.s.d.'s involving l.s. planes.

### Fractional atomic coordinates and isotropic or equivalent isotropic displacement parameters (Å^2^)

**Table:**

	*x*	*y*	*z*	*U*_iso_*/*U*_eq_	
Cu1	0.32501 (3)	1.00767 (2)	0.977106 (17)	0.03806 (10)	
N1	0.1622 (2)	1.08781 (15)	0.95744 (11)	0.0313 (4)	
N2	0.2672 (3)	0.89079 (19)	1.06492 (15)	0.0491 (6)	
N3	0.5005 (2)	0.93672 (19)	1.00252 (14)	0.0480 (6)	
O4	0.3191 (2)	0.93362 (17)	0.88490 (11)	0.0498 (5)	
O2	0.29386 (19)	1.31490 (16)	1.05070 (12)	0.0512 (5)	
O1	0.37076 (18)	1.14608 (15)	1.03256 (11)	0.0424 (4)	
O3	−0.05190 (19)	1.07490 (16)	1.05400 (12)	0.0474 (5)	
H3	−0.0961	1.1082	1.0236	0.071*	
C1	0.2783 (3)	1.2178 (2)	1.03223 (14)	0.0371 (6)	
C2	0.1394 (3)	1.17825 (19)	1.00763 (13)	0.0319 (5)	
H28	0.0912	1.2379	0.9832	0.038*	
C3	0.0596 (3)	1.1395 (2)	1.07501 (15)	0.0368 (6)	
H29	0.1194	1.0925	1.1037	0.044*	
C4	0.0177 (3)	1.2351 (3)	1.12313 (16)	0.0510 (7)	
H21A	−0.0285	1.2076	1.1651	0.077*	
H21B	−0.0409	1.2830	1.0965	0.077*	
H21C	0.0960	1.2750	1.1382	0.077*	
C5	0.0856 (2)	1.0765 (2)	0.90139 (14)	0.0325 (5)	
H11	0.0125	1.1237	0.8978	0.039*	
C6	0.1028 (2)	0.9982 (2)	0.84450 (12)	0.0363 (5)	
C7	0.2228 (3)	0.9366 (2)	0.83774 (15)	0.0415 (6)	
C8	0.2402 (3)	0.8722 (3)	0.77391 (16)	0.0545 (8)	
H18	0.3199	0.8335	0.7678	0.065*	
C9	0.1453 (4)	0.8654 (3)	0.72205 (16)	0.0560 (8)	
H19	0.1615	0.8224	0.6813	0.067*	
C10	0.0215 (3)	0.9221 (2)	0.72803 (15)	0.0452 (6)	
C11	−0.0016 (3)	0.9893 (2)	0.78988 (13)	0.0397 (6)	
C12	−0.1269 (3)	1.0408 (2)	0.79527 (17)	0.0495 (7)	
H27	−0.1453	1.0845	0.8354	0.059*	
C13	−0.2234 (4)	1.0284 (3)	0.74247 (19)	0.0588 (8)	
H26	−0.3055	1.0640	0.7475	0.071*	
C14	−0.2000 (4)	0.9635 (3)	0.68174 (18)	0.0621 (9)	
H25	−0.2659	0.9556	0.6464	0.074*	
C15	−0.0795 (4)	0.9115 (3)	0.67446 (17)	0.0572 (8)	
H15	−0.0634	0.8685	0.6337	0.069*	
C16	0.1466 (4)	0.8711 (3)	1.0941 (2)	0.0660 (9)	
H17	0.0701	0.8976	1.0710	0.079*	
C17	0.1333 (6)	0.8119 (3)	1.1577 (3)	0.0965 (13)	
H22	0.0488	0.7999	1.1777	0.116*	
C18	0.2463 (6)	0.7710 (4)	1.1912 (3)	0.1063 (14)	
H36	0.2387	0.7302	1.2336	0.128*	
C19	0.3693 (5)	0.7909 (3)	1.1617 (3)	0.0883 (12)	
H37	0.4468	0.7665	1.1849	0.106*	
C20	0.3776 (4)	0.8483 (2)	1.09632 (19)	0.0579 (8)	
C21	0.5052 (3)	0.8664 (2)	1.05811 (19)	0.0524 (7)	
C22	0.6242 (4)	0.8126 (3)	1.0751 (2)	0.0701 (10)	
H33	0.6271	0.7624	1.1131	0.084*	
C23	0.7371 (4)	0.8343 (3)	1.0354 (3)	0.0775 (11)	
H23	0.8169	0.7986	1.0468	0.093*	
C24	0.7348 (3)	0.9067 (3)	0.9799 (3)	0.0719 (10)	
H24	0.8118	0.9222	0.9532	0.086*	
C25	0.6136 (3)	0.9570 (3)	0.9642 (2)	0.0634 (9)	
H32	0.6097	1.0066	0.9259	0.076*	

### Atomic displacement parameters (Å^2^)

**Table:**

	*U*^11^	*U*^22^	*U*^33^	*U*^12^	*U*^13^	*U*^23^
Cu1	0.03383 (16)	0.04027 (16)	0.04008 (17)	0.00393 (14)	−0.00421 (14)	0.00230 (15)
N1	0.0301 (10)	0.0347 (10)	0.0291 (11)	0.0002 (9)	0.0011 (9)	−0.0009 (8)
N2	0.0536 (15)	0.0405 (13)	0.0531 (16)	−0.0017 (11)	−0.0030 (13)	0.0083 (12)
N3	0.0409 (13)	0.0384 (13)	0.0647 (17)	0.0035 (10)	−0.0093 (12)	0.0003 (11)
O4	0.0456 (11)	0.0590 (12)	0.0448 (12)	0.0165 (10)	−0.0016 (11)	−0.0084 (9)
O2	0.0434 (11)	0.0466 (11)	0.0636 (14)	−0.0096 (9)	0.0033 (10)	−0.0158 (10)
O1	0.0341 (9)	0.0460 (10)	0.0470 (12)	−0.0025 (8)	−0.0092 (9)	−0.0015 (9)
O3	0.0356 (10)	0.0553 (12)	0.0514 (13)	−0.0103 (9)	0.0020 (9)	0.0047 (10)
C1	0.0398 (13)	0.0424 (13)	0.0291 (13)	−0.0075 (11)	0.0053 (11)	−0.0008 (11)
C2	0.0348 (12)	0.0333 (12)	0.0275 (12)	0.0016 (10)	−0.0021 (10)	−0.0008 (9)
C3	0.0339 (13)	0.0442 (14)	0.0323 (13)	−0.0029 (11)	0.0013 (11)	0.0017 (11)
C4	0.0536 (18)	0.0621 (19)	0.0374 (15)	0.0016 (15)	0.0060 (14)	−0.0050 (14)
C5	0.0326 (12)	0.0360 (12)	0.0288 (12)	0.0016 (10)	−0.0001 (10)	0.0002 (10)
C6	0.0456 (13)	0.0364 (12)	0.0270 (11)	0.0003 (13)	0.0012 (10)	−0.0009 (11)
C7	0.0486 (15)	0.0413 (14)	0.0346 (14)	0.0009 (12)	0.0065 (12)	−0.0036 (11)
C8	0.0637 (19)	0.0570 (17)	0.0428 (17)	0.0105 (15)	0.0104 (15)	−0.0090 (14)
C9	0.074 (2)	0.0580 (16)	0.0354 (15)	−0.0053 (16)	0.0078 (15)	−0.0131 (13)
C10	0.0586 (16)	0.0469 (15)	0.0299 (13)	−0.0123 (13)	0.0007 (13)	−0.0025 (11)
C11	0.0510 (14)	0.0392 (13)	0.0291 (11)	−0.0082 (13)	−0.0017 (11)	0.0002 (11)
C12	0.0552 (16)	0.0542 (15)	0.0392 (15)	−0.0003 (13)	−0.0115 (13)	−0.0065 (12)
C13	0.0596 (17)	0.065 (2)	0.0515 (17)	−0.0031 (15)	−0.0187 (15)	−0.0011 (15)
C14	0.076 (2)	0.0662 (18)	0.0440 (16)	−0.0210 (16)	−0.0221 (16)	0.0030 (14)
C15	0.079 (2)	0.0582 (17)	0.0344 (15)	−0.0206 (16)	−0.0067 (15)	−0.0039 (13)
C16	0.074 (2)	0.0503 (17)	0.074 (2)	−0.0007 (16)	0.0131 (19)	0.0167 (16)
C17	0.104 (3)	0.085 (3)	0.101 (3)	0.002 (2)	0.034 (3)	0.038 (2)
C18	0.128 (3)	0.098 (3)	0.092 (3)	0.009 (3)	0.010 (3)	0.049 (2)
C19	0.100 (3)	0.080 (2)	0.084 (2)	0.015 (2)	−0.007 (2)	0.035 (2)
C20	0.0753 (19)	0.0387 (14)	0.0597 (18)	0.0037 (15)	−0.0113 (17)	0.0086 (14)
C21	0.0575 (17)	0.0352 (14)	0.0645 (18)	0.0071 (13)	−0.0215 (15)	−0.0033 (14)
C22	0.076 (2)	0.0502 (17)	0.084 (2)	0.0146 (17)	−0.029 (2)	−0.0012 (17)
C23	0.060 (2)	0.063 (2)	0.109 (3)	0.0182 (17)	−0.031 (2)	−0.020 (2)
C24	0.0489 (17)	0.0566 (18)	0.110 (3)	0.0058 (15)	−0.010 (2)	−0.015 (2)
C25	0.0492 (17)	0.0481 (16)	0.093 (2)	0.0030 (13)	−0.0050 (18)	−0.0026 (16)

### Geometric parameters (Å, °)

**Table:**

Cu1—O4	1.925 (2)	C8—H18	0.9300
Cu1—N1	1.926 (2)	C9—C10	1.417 (5)
Cu1—N3	2.005 (2)	C9—H19	0.9300
Cu1—O1	2.0236 (18)	C10—C15	1.415 (4)
Cu1—N2	2.231 (3)	C10—C11	1.422 (4)
N1—C5	1.292 (3)	C11—C12	1.400 (4)
N1—C2	1.457 (3)	C12—C13	1.376 (4)
N2—C16	1.338 (4)	C12—H27	0.9300
N2—C20	1.345 (4)	C13—C14	1.390 (5)
N3—C21	1.337 (4)	C13—H26	0.9300
N3—C25	1.352 (4)	C14—C15	1.363 (5)
O4—C7	1.295 (4)	C14—H25	0.9300
O2—C1	1.240 (3)	C15—H15	0.9300
O1—C1	1.269 (3)	C16—C17	1.384 (5)
O3—C3	1.415 (3)	C16—H17	0.9300
O3—H3	0.8200	C17—C18	1.376 (7)
C1—C2	1.533 (4)	C17—H22	0.9300
C2—C3	1.548 (4)	C18—C19	1.362 (7)
C2—H28	0.9800	C18—H36	0.9300
C3—C4	1.522 (4)	C19—C20	1.397 (5)
C3—H29	0.9800	C19—H37	0.9300
C4—H21A	0.9600	C20—C21	1.469 (5)
C4—H21B	0.9600	C21—C22	1.390 (5)
C4—H21C	0.9600	C22—C23	1.366 (6)
C5—C6	1.428 (3)	C22—H33	0.9300
C5—H11	0.9300	C23—C24	1.352 (6)
C6—C7	1.417 (4)	C23—H23	0.9300
C6—C11	1.452 (3)	C24—C25	1.384 (5)
C7—C8	1.425 (4)	C24—H24	0.9300
C8—C9	1.346 (5)	C25—H32	0.9300
			
O4—Cu1—N1	92.62 (8)	C9—C8—H18	118.8
O4—Cu1—N3	91.77 (9)	C7—C8—H18	118.8
N1—Cu1—N3	174.65 (9)	C8—C9—C10	121.6 (3)
O4—Cu1—O1	147.49 (9)	C8—C9—H19	119.2
N1—Cu1—O1	82.08 (8)	C10—C9—H19	119.2
N3—Cu1—O1	92.57 (9)	C15—C10—C9	121.3 (3)
O4—Cu1—N2	109.51 (10)	C15—C10—C11	119.8 (3)
N1—Cu1—N2	104.07 (9)	C9—C10—C11	118.9 (3)
N3—Cu1—N2	77.29 (10)	C12—C11—C10	117.3 (3)
O1—Cu1—N2	102.88 (9)	C12—C11—C6	123.7 (2)
C5—N1—C2	119.8 (2)	C10—C11—C6	118.9 (3)
C5—N1—Cu1	126.40 (17)	C13—C12—C11	121.5 (3)
C2—N1—Cu1	113.27 (15)	C13—C12—H27	119.3
C16—N2—C20	119.4 (3)	C11—C12—H27	119.3
C16—N2—Cu1	129.6 (2)	C12—C13—C14	121.0 (3)
C20—N2—Cu1	110.3 (2)	C12—C13—H26	119.5
C21—N3—C25	119.3 (3)	C14—C13—H26	119.5
C21—N3—Cu1	119.1 (2)	C15—C14—C13	119.4 (3)
C25—N3—Cu1	121.6 (2)	C15—C14—H25	120.3
C7—O4—Cu1	127.02 (18)	C13—C14—H25	120.3
C1—O1—Cu1	114.08 (16)	C14—C15—C10	120.9 (3)
C3—O3—H3	109.5	C14—C15—H15	119.5
O2—C1—O1	124.4 (2)	C10—C15—H15	119.5
O2—C1—C2	119.6 (2)	N2—C16—C17	121.3 (4)
O1—C1—C2	116.1 (2)	N2—C16—H17	119.3
N1—C2—C1	106.54 (19)	C17—C16—H17	119.3
N1—C2—C3	111.06 (19)	C18—C17—C16	119.4 (5)
C1—C2—C3	108.7 (2)	C18—C17—H22	120.3
N1—C2—H28	110.1	C16—C17—H22	120.3
C1—C2—H28	110.1	C19—C18—C17	119.5 (4)
C3—C2—H28	110.1	C19—C18—H36	120.3
O3—C3—C4	111.7 (2)	C17—C18—H36	120.3
O3—C3—C2	110.6 (2)	C18—C19—C20	119.1 (4)
C4—C3—C2	112.0 (2)	C18—C19—H37	120.4
O3—C3—H29	107.4	C20—C19—H37	120.4
C4—C3—H29	107.4	N2—C20—C19	121.1 (4)
C2—C3—H29	107.4	N2—C20—C21	116.2 (3)
C3—C4—H21A	109.5	C19—C20—C21	122.7 (4)
C3—C4—H21B	109.5	N3—C21—C22	120.3 (4)
H21A—C4—H21B	109.5	N3—C21—C20	115.7 (3)
C3—C4—H21C	109.5	C22—C21—C20	123.9 (3)
H21A—C4—H21C	109.5	C23—C22—C21	119.3 (4)
H21B—C4—H21C	109.5	C23—C22—H33	120.4
N1—C5—C6	126.0 (2)	C21—C22—H33	120.4
N1—C5—H11	117.0	C24—C23—C22	121.2 (3)
C6—C5—H11	117.0	C24—C23—H23	119.4
C7—C6—C5	121.3 (2)	C22—C23—H23	119.4
C7—C6—C11	120.2 (2)	C23—C24—C25	117.5 (4)
C5—C6—C11	118.3 (2)	C23—C24—H24	121.3
O4—C7—C6	125.5 (2)	C25—C24—H24	121.3
O4—C7—C8	116.7 (3)	N3—C25—C24	122.4 (4)
C6—C7—C8	117.8 (3)	N3—C25—H32	118.8
C9—C8—C7	122.4 (3)	C24—C25—H32	118.8
			
C1—C2—C3—C4	71.0 (3)		

### Hydrogen-bond geometry (Å, °)

**Table:**

*D*—H···*A*	*D*—H	H···*A*	*D*···*A*	*D*—H···*A*
O3—H3···O2^i^	0.82	1.99	2.808 (3)	178
C5—H11···O2^i^	0.93	2.49	3.311 (3)	147
C12—H27···O2^i^	0.93	2.50	3.431 (4)	174
C14—H25···O3^ii^	0.93	2.52	3.445 (4)	177

Symmetry codes: (i) *x*−1/2, −*y*+5/2, −*z*+2; (ii) −*x*−1/2, −*y*+2, *z*−1/2.
